# The Use of Methylene Blue during Liver Transplantation for Vasoplegia

**DOI:** 10.1155/2021/6610754

**Published:** 2021-06-23

**Authors:** Paul Harding, Thomas Nicholas, Cale Kassel

**Affiliations:** ^1^University of Nebraska Medical Center—College of Medicine, Nebraska Medical Center, Omaha 68198-5520, NE, USA; ^2^University of Nebraska Medical Center—Department of Anesthesiology, Nebraska Medical Center, Omaha 68198-7541, NE, USA

## Abstract

The use of methylene blue for vasoplegia in cardiac cases with cardiopulmonary bypass, septic shock, and acute liver failure is well documented. Use of MB for liver transplantation has been largely limited to case reports. We describe three separate liver transplantation patients with significant hypotension following reperfusion. Administration of methylene blue to each patient resulted in a significant decrease in vasopressor medication and two patients weaned completely. We argue that the use of MB should be considered as a treatment option for refractory hypotension.

## 1. Introduction

Traditionally used in the treatment of methemoglobinemia, methylene blue (MB) use for refractory hypotension was first described in sepsis and cardiac surgery [1, 2]. In liver transplantation (LT), the consequences of postreperfusion syndrome (PRS) and ischemia-reperfusion injury can be challenging to manage for anesthesiologists. The use of MB in LT can be a useful medication in management refractory hypotension and vasoplegia. We present three cases of the use of MB in LT as a potential therapy and suggest a simple stepwise approach to vasoplegia in LT.

## 2. Case Series

### 2.1. Case One

A 63-year-old female presented for a LT from a donation after brain death (DBD) donor. Her first LT was in 1997 for primary sclerosing cholangitis. Since that time, she developed worsening hepatic encephalopathy, refractory ascites, portal hypertension, and hepatorenal syndrome (not requiring dialysis). Her MELD score at the time of her transplant was 36. She also was found to have a left ventricular outflow tract (LVOT) gradient on dobutamine stress echocardiography (DSE). Given her previous LT and DSE findings, we elected to utilize veno-venous bypass with a portal shunt for her LT.

Following induction, she was started on phenylephrine infusion at 50 mcg/min and norepinephrine infusion at 2 mcg/min was added for blood pressure support. The phenylephrine was increased to 100 mcg/min and norepinephrine to 4 mcg/min with incremental boluses during dissection.

**Figure 1:**
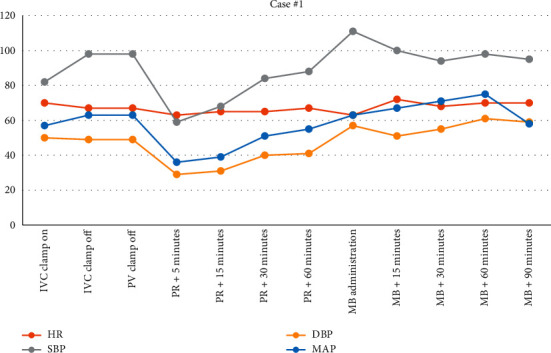
Vital signs from IVC clamp until 90 minutes following MB administration. IVC, inferior vena cava; PV, portal vein; PR, postreperfusion; MB, methylene blue.

**Figure 2:**
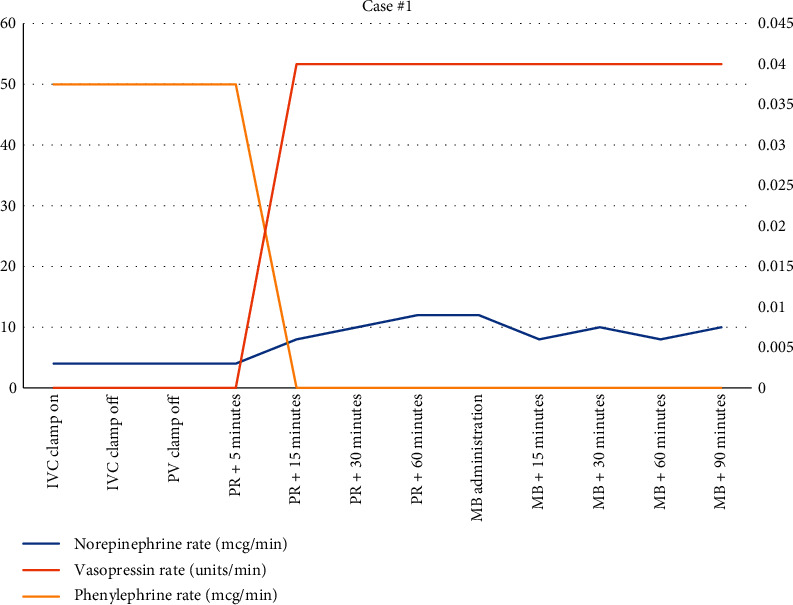
Vasoactive medication use during case #1.

The duration of anhepatic phase was 50 minutes. She tolerated initiation of veno-venous bypass as well as total caval occlusion without significant changes in hemodynamics. Upon reperfusion, there was marked hypotension secondary to combined hypovolemia and low SVR (283 Dynes/sec/cm^5^) Left ventricular ejection fraction of 65–70% was confirmed by transesophageal echocardiography. Although the patient had a preoperative diagnosis of LVOT obstruction, it was felt that the patient did not have clinically significant obstruction after interrogation with 2D and color flow Doppler. Though blood loss was significant throughout the case (>6 L estimated), she was resuscitated adequately as assessed by TEE. Her worsening hypotension required continued phenylephrine at 50 mcg/min, norepinephrine up to 20 mcg/min, and vasopressin at 0.04 units/min. As we were unable to maintain our goal mean arterial pressure (MAP) of 65 mmHg, the decision was made to administer methylene blue (1 mg/kg). As seen in Figure 1, her hemodynamics improved and no longer required escalating doses of vasoactive medications.  Figure 2shows that though she remained on vasopressin (0.04 units/min) and norepinephrine (8 mcg/min) when transferred to the ICU, her clinical picture improved. Her pH before arriving in the ICU was 7.41 (from 7.26) and lactic acid decreased from 7.8 to 5.5 within 1 hour of giving methylene blue.

### 2.2. Case Two

**Figure 3:**
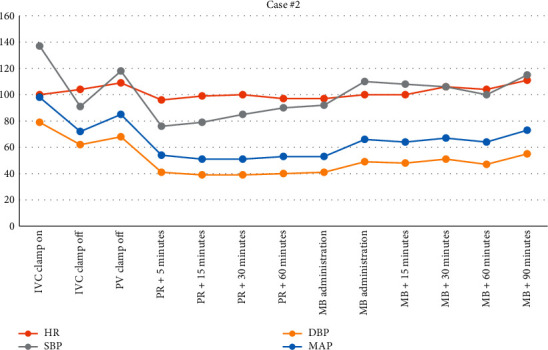
Vital signs from IVC clamp until 90 minutes following MB administration.

**Figure 4:**
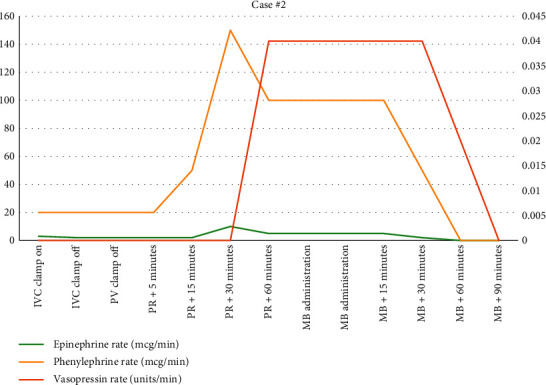
Vasoactive medication use during case #2.

A 49-year-old female with a history of nonalcoholic steatohepatitis (NASH) cirrhosis, type II diabetes mellitus, hypertension, and chronic obstructive pulmonary disease underwent LT. Her Model for End-Stage Liver Disease (MELD) score was 25 before transplant. She received a donation after cardiac death (DCD) organ for her LT. During the preanhepatic phase, she required intermittent bolus doses of phenylephrine (500 mcg total). Seven minutes before IVC clamping, she was started on epinephrine infusion at 3 mcg/min and phenylephrine infusion at 20 mcg/min for hypotension. During the anhepatic phase (74 minutes), she required similar doses of epinephrine and phenylephrine. After reperfusion, the epinephrine infusion was increased to 10 mcg/min and phenylephrine dose was increased to 150 mcg/min. Vasopressin infusion was also added 45 minutes after reperfusion at 0.04 units/min. The patient remained tachycardic and hypotensive despite these efforts. Transesophageal echocardiography (TEE) revealed a hyperdynamic state with a low SVR (416 dynes/sec/cm^5^). Additionally, normal cardiac filling volumes were present, indicating adequate resuscitation even with a MAP less than 65 mmHg. At this time, we elected to administer methylene blue (MB) for refractory hypotension. She was given a total of 100 mg over 20 minutes. Figures 3 and 4 show the effects of MB on her blood pressure and we were able to rapidly wean down her vasopressor requirements. Upon transfer to the ICU, she required no vasoactive medications.

### 2.3. Case Three

**Figure 5:**
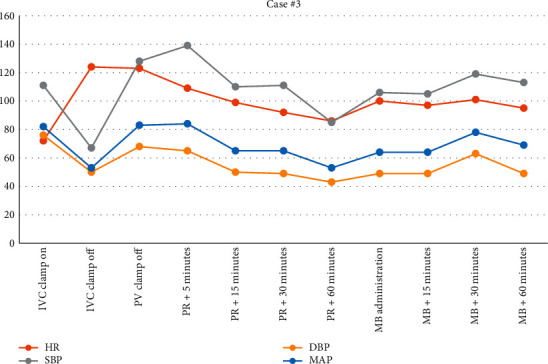
Vital signs from IVC clamp until 90 minutes following MB administration.

**Figure 6:**
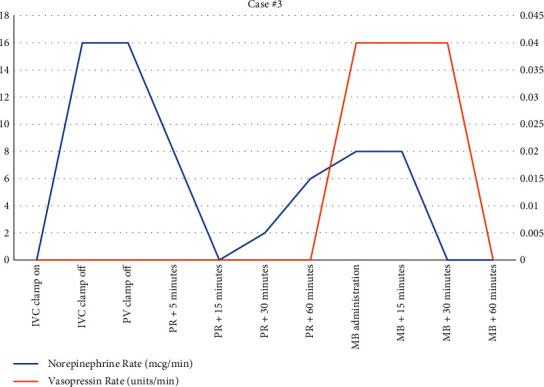
Vasoactive medication use during case #3.

The final case involved a 65-year-old male with hepatocellular carcinoma presenting for LT. Past medical history included hypertension, hyperlipidemia, insulin-dependent type 2 diabetes mellitus, and hypothyroidism. From induction to the anhepatic phase, he remained remarkably stable. However, during the anhepatic phase, the patient became hypotensive with underfilled ventricles requiring norepinephrine infusion up to 16 mcg/min. Initially, the patient was stable after, but became increasingly hypotensive despite volume resuscitation. Systemic vascular resistance was low (653 Dynes/sec/cm^5^) and vasopressin was then added at 0.04 units/min in addition to norepinephrine at 16 mcg/min. Blood loss was significant (>7 L was estimated), but the patient received a significant amount of blood products that included 22 units of plasma, 8 units of red blood cells, and 5.7 L of crystalloid. His volume status was watched throughout the procedure utilizing TEE. Given that he received a donation after cardiac death (DCD) organ and was requiring significant vasoactive medication to support a MAP of 65 mmHg, the decision was made to administer MB (50 mg). This improved his hemodynamics quickly as noted in Figure 5.  Figure 6shows that 22 minutes after administration of MB, the norepinephrine infusion was discontinued. Vasopressin was discontinued 32 minutes after MB administration.

## 3. Discussion

The use of MB for refractory hypotension continues to be an area of interest during liver transplantation. Based on evidence from sepsis, cardiac surgery, and acute liver failure, successful use of MB in LT has been described over the years in various case reports [3–5]. Though limited to case reports, a growing body of evidence suggests MB can be useful for hypotension refractory to standard therapies during LT.

Postreperfusion syndrome (PRS) was first described in 1987 as a “transient, profound cardiovascular collapse” following reperfusion of the new liver [6]. This included decreases in mean arterial pressure (MAP) and systemic vascular resistance (SVR) with increased pulmonary artery pressure (PAP) and pulmonary capillary wedge pressure (PCWP). Hilmi expanded on the definition classifying PRS as either mild or significant [7]. A hallmark of significant PRS is the need for vasopressor infusion intraoperatively. The incidence of PRS varies widely from as low as 8% to rates as high as 77% [8, 9].

While PRS has a distinct set conditions immediately following graft reperfusion, vasoplegia is often harder to describe. A key element of vasoplegia is low SVR, often in the setting of normal or increased cardiac output (CO) [10]. Commonly associated with cardiac surgery, it can be seen in noncardiac surgery, sepsis, and perioperative use of angiotensin-converting enzyme inhibitors (ACE-I) [11]. The incidence of vasoplegia in LT is hard discerned owing to the lack of universally accepted criteria and overlap with PRS. Release of nitric oxide, carbon monoxide, and hydrogen sulfide all contribute to vasodilation as well as a deficiency of vasopressin [12].

A heterocyclic aromatic dye, MB, can be used as a treatment of methemoglobin or as an indicator dye. More recently, understanding how MB inhibits nitric oxide synthase and reduces the production of nitric oxide (NO) created new opportunities for MB use in perioperative care of LT patients.

One of the first published reports of MB use in LT was by Koelzow et al. [13] in 2002. In their study, they described the hemodynamic effects of PRS described above and postulated the use of MB could be used to improve specific hemodynamic parameters after reperfusion. After randomizing 36 patients to receive MB or normal saline before graft reperfusion, they compared MAP, cardiac index (CI), and systemic vascular resistance (SVR). There was not a significant difference in SVR between the two groups. Serum lactate was also significantly lower in the MB group at 1 hour compared to the control group. Graft function and length of stay were not statistically different between the MB group and control group. Even with the small number of patients, this study offered insight into how MB could be a valuable tool in PRS.

Fukazawa and Pretto retrospectively reviewed 715 LT patients and the propensity score matched those who received MB to those who did not [14]. A total of 105 patients received MB and were largely similar to the control group, except for the MB group being older (55.5 ± 0.9 vs. 53.1 ± 0.8 years, *p* = 0.026). The rate of PRS was similar between the two groups (55.7% vs. 55.8%, *p* = 0.993). Overall, there were no significant differences in percent changes in MAP after reperfusion, use of vasopressors within 30 minutes of reperfusion, or postreperfusion vasopressor use. Transfusion requirements were not significantly different either.

Other case reports on the use of MB in LT are limited, but have shown success in improving vasoplegia. Cao et al. reported the use of MB for vasoplegia following reperfusion of the liver. The patient required increasing doses of norepinephrine and despite this their SVR remained low (369 dynes/s/cm^2^). A single dose of 0.5 mg/kg of MB was administered with improvement in blood pressure and SVR. Ultimately, norepinephrine was weaned off within three hours of arriving in the ICU [5]. Another case report from Levin et al. described a similar use of MB in a LT [2]. Despite the use of three different vasopressor medications, TEE showed an elevated CO and low SVR. A dose of MB at 2 mg/kg (over 30 minutes) followed by an infusion at 0.5 mg/kg/h improved the patient's hemodynamics improved promptly and they were able to wean two of the three vasopressor medications. Finally, Daemen-Gubbels et al. described three patients who received MB during LT at various stages in the procedure [1]. Two of the three patients received 100 mg doses of MB and the third was given 1.5 mg/kg all with good response and improvement in hemodynamics.

All three of our patients described had low SVR based on TEE findings, consistent with some degree of vasoplegia. In fact, most of the challenges of hypotension in these patients were during the neohepatic period after the period when PRS would be the concern. Wagener et al. noted that routine use of MB in LT did not prevent postreperfusion hypotension, decrease transfusion requirements, or decrease vasopressor use. However, those that received MB were at the provider's discretion and typically given before reperfusion, not after reperfusion [12]. Most case reports including ours report administration of MB after reperfusion. Perhaps, as Cao and Tao noted in an editorial response, MB is better suited for the treatment of VS not PRS [15]. Specifically, VS and the association with increased NO may explain why MB works better when administered following reperfusion [16]. As many of the other case reports noted, initial reperfusion was challenging but the hypotension and vasopressor requirements continued past the initial 5 minutes. This differs from the conclusion of the Koelzow et al.'s study which was “a single bolus of MB has limited capacity to prevent hypotension immediately following portal revascularization and reperfusion” [13]. Indeed, we would not argue for the routine use of MB in LT patients. Evidence to date does not support routine use of MB, and further studies to identify patients in which MB would be beneficial are needed [17].

How best then should anesthesiologists utilize MB during LT? We argue for a stepwise approach to vasoplegia in LT patients to rule out common causes of hypotension before utilizing MB. Review of laboratory findings to should focus on ensuring appropriate hemoglobin and calcium levels. Utilization of a thromboelastogram (TEG) to guide transfusion and assess coagulation status is also useful [18]. Use of TEE can be helpful to guide assessment volume, rule out pulmonary embolism, evaluate ventricular function, and assess SVR [19]. As described in our cases, we recognized that volume status (as measured by CO) was adequate, yet SVR remained low despite multiple vasopressors. Vasopressors such as norepinephrine, epinephrine, or phenylephrine remain the standard treatment for VS, but some cases remain refractory to treatment. The choice of vasoactive medication should be based on clinical findings, but vasopressin may be useful as a first line agent given the relative deficiency in ESLD [12]. After review of laboratory and TEG findings and TEE assessment, if the use of vasoactive medications continues to increase or fail to improve hemodynamic parameters, MB should be considered if there are no contraindications.

The use of MB remains a useful option in vasoplegia and PRS for transplant anesthesiologists. We argue in favor of establishing protocols for their use during LT. By standardizing the approach to the use of MB, other causes of hypotension can be ruled out before administration. Despite the benefits described in case studies, there remains a lack of randomized controlled trials describing their use in LT.

## Data Availability

The data used to support the findings of this study are included within the article.
